# Fc-gamma receptors type3A (rs396991) genotyping for predicting infliximab efficacy and immunogenicity in ulcerative colitis: An observational study of Iraqi cohort

**DOI:** 10.1097/MD.0000000000046119

**Published:** 2026-02-28

**Authors:** Ahmad K. Al-Jalehawi, Samer Imad Mohammed

**Affiliations:** a Clinical Pharmacy Department, College of Pharmacy, University of Baghdad, Baghdad, Iraq; b Clinical Pharmacy Department, College of Pharmacy, University of Alkafeel, Najaf, Iraq.

**Keywords:** antidrug antibodies, FCGR3A polymorphism, infliximab, pharmacogenetics, ulcerative colitis

## Abstract

Anti-tumor necrosis factor treatments for inflammatory bowel disease face challenges like primary nonresponse and secondary loss of response, often due to antidrug antibodies that increase drug clearance. The Fc-gamma receptors type3A (FCGR3A) (rs396991) polymorphism affects infliximab pharmacokinetics and immunogenicity. This study investigates its influence on trough levels, anti-infliximab antibody development, and clinical outcomes in Iraqi ulcerative colitis (UC) patients. This single-center study involved patients on maintenance infliximab therapy who were enrolled. Serum infliximab trough levels and anti-infliximab antibodies (antibodies to infliximab) (free and total) were measured using enzyme-linked immunosorbent assay. Genotyping of the FCGR3A rs396991 polymorphism was performed via polymerase chain reaction amplification and Sanger sequencing. The partial Mayo score assessed disease activity. The significance level of statistics was *P* < .05. Among 43 patients, those with the CC genotype achieved target infliximab trough levels more frequently (55.6%) than AA (21.1%) or AC (0%) genotypes (*P* = .005). Median infliximab levels were highest in CC carriers (3.41 µg/mL, *P* = .022). The AC genotype had a significantly higher prevalence of total anti-infliximab antibodies (53.3%) compared to CC (22.2%) and AA (10.5%) groups (*P* = .02). Logistic regression confirmed the CC genotype positive association with therapeutic drug levels and lower antibody positivity, while the AC genotype correlated with increased immunogenicity. The FCGR3A rs396991 CC genotype is significantly associated with improved infliximab pharmacokinetics and reduced immunogenicity in UC patients. These findings highlight the potential of FCGR3A genotyping to guide personalized therapeutic strategies and optimize clinical outcomes in UC.

## 1. Introduction

Despite the demonstrated efficacy of anti-tumor necrosis factor (TNF) therapies in various inflammatory conditions, including inflammatory bowel disease (IBD).^[1–3]^ A significant percentage of patients either fails to respond to treatment (primary nonresponse) or loses the effectiveness of treatment over time (secondary loss of response, LOR). Primary nonresponse affects 10% to 30% of patients, while LOR occurs at rates of up to 46%.^[4,5]^ These challenges underscore the inherent complexities in predicting and sustaining treatment outcomes in anti-TNF-treated populations. The presence of antibody toward infliximab (IFX) might be as high as 70% in Iraqi population.^[6]^

The mechanisms underlying LOR are multifaceted, with a prominent contributing factor being the development of antidrug antibodies (ADA), particularly antibodies to infliximab (ATI). This immunogenic response can lead to expedited drug clearance and reduced serum drug concentrations, directly compromising therapeutic efficacy.^[7,8]^ Beyond immunogenicity, subtherapeutic drug levels can also result from other factors such as rapid intrinsic drug clearance or inadequate dosing.^[7,9]^ The half-life of immunoglobulin G (IgG) antibodies, to which infliximab belongs, is influenced by various factors.^[7,9]^

This variation emphasizes how crucial it is to create individualized treatment plans. Finding biomarkers that may predict therapeutic responses is a potential application of pharmacogenetics. In particular, because of its substantial impact on antibody binding affinity and immune complex interactions, the rs396991 polymorphism in the Fc-gamma receptors type3A (FCGR3A) gene, which codes for the Fcγ receptor IIIa, is a promising candidate.^[10,11]^

The rs396991 polymorphism significantly influences infliximab pharmacokinetics and immunogenicity, which results in the encoding of 2 distinct amino acids at position 158: phenylalanine (F) and valine (V). The genotypic variations correspond to AA coding for the FF homozygous phenotype, AC representing the heterozygous VF, and CC coding for the VV homozygous phenotype.^[12]^ The valine (V) allele confers high affinity for IgG1 and IgG3 subclasses, whereas the phenylalanine (F) allele results in low-affinity binding^[13]^ This polymorphism is found to be a potential biomarker for disease activity.^[14]^

Further evidence showing that carriers of the variant FCGR3A allele exhibit reduced clinical response to infliximab at multiple time points during treatment and have lower median infliximab trough levels (IFX TL). These patients also demonstrated a higher likelihood of ADA production, reinforcing the impact of FCGR3A polymorphisms on both pharmacokinetics and immunogenicity in ulcerative colitis (UC) and Crohn’s disease (CD) alike.^[13]^ Pediatric and adult studies demonstrate that the V allele correlates with reduced clinical response at induction (*P* = .004), 22 weeks (*P* = .001), and 52 weeks (*P* = .01), lower IFX TL, and higher ATI production. Specifically, CC (VV) homozygotes exhibit a 37.5% ADA incidence versus 5% in FF carriers (*P* = .004) and require more frequent dose intensification.^[15]^

Conversely, some data suggest CC genotype may enhance biological response in CD, evidenced by greater C-reactive protein reduction.^[16]^ These divergent findings highlight the polymorphism complex role in treatment efficacy and the need for population-specific validation. Despite some ongoing genetics research investigating the role of pharmacogenetics in biological treatments,^[17–19]^ no study has been conducted on the effect of FCGR3A in the Iraqi population.

This study aims to fill the significant gap in pharmacogenetic research on Iraqi patients suffering from UC, by examining the association between rs396991 genotypes and key clinical factors such as IFX TL, ATI development, and treatment outcomes.

## 2. Methods

This study is a part of a PhD thesis. A total of 43 patients diagnosed with UC participated in the study after providing informed consent. These patients were attending a specialized hospital for gastroenterology and hepatology for their scheduled IFX doses. The inclusion criteria required participants to be over 18 years of age and in the maintenance phase of IFX therapy, irrespective of their concurrent medications or surgical history. Serum samples were collected by taking blood samples from forearm before the dose was administered. Enzyme-linked immunosorbent assays (sandwich method) was performed based on the kit protocol to measure IFX TL, as well as free and total ATI, using a kit from Matriks Biotek, Ankara,Türkiye. Disease activity was measured using the partial Mayo score for UC activity assessment.

### 2.1. Ethical approval

Ethical approval was granted by the Health Directorate, Ministry of Health (No.7798) and appropriate informed consent was taken from the patients.

### 2.2. Deoxyribonucleic acid (DNA) extraction and sequence

Primer was designed to perform genetic sequencing on the FCGR3A gene for a larger analysis with forward and backward primers (764 5′-TGATCACCAGGAGGGAACCACATATGAA-3′ 791) and (111 5′-ACCCTATTCACCTGAGGTGTCACAGCTG-3′ 1084), respectively.

The extraction was caried on using DNA extraction kit from Favorgen Biotech Corp., Pingtung, Taiwan. The DNA was amplified using a primer pair at temperatures ranging from 55°C to 65°C, with 60°C identified as the most effective to determine the optimal annealing temperature. Polymerase chain reaction amplifications were conducted using GoTaq Green Master Mix (Promega Corporation, Madison), primers, nuclease-free water, and template DNA, following a thermal cycling program. The polymerase chain reaction products were then confirmed by agarose gel electrophoresis and visualized using gel imaging. The genetic sequence was obtained through Sanger sequencing conducted by Macrogen Corporation (Seoul, South Korea) .

### 2.3. Statistical analysis

Statistical analysis performed using SPSS (version 26; IBM Corp., Chicago). IFX target TL for clinical improvement was considered (≥3 µg/mL), and free and total ATI were quantitatively and qualitatively assessed based on the kit manual. Multivariate logistic regression was performed to see the effect of each genotype on the IFX, free, and total ATI. nonparametric analysis (Chi-square, fisher’s exact and Kruskal–Wallis) test was used for comparison of different patient groups, and a significant value was set as (*P* < .05).

## 3. Results

The study cohort consisted of 43 patients with UC, as demonstrated in Table 1, with a mean age of 34.5 ± 11.0 years, and a slight male predominance (60.5%). The average body mass index was 25.0 ± 4.89, indicating a generally normal to slightly overweight population. Patients had a mean disease duration of 4.87 ± 4.14 years, and had been receiving infliximab therapy for an average of 114.12 ± 85.34 weeks, reflecting a population with established disease and prolonged biologic treatment. Nearly all patients were on azathioprine (95.35%) and mesalamine (93.02%), suggesting intensive combination therapy. Despite this, 60.46% of patients exhibited active disease, whereas 39.54% were in remission. Notably, only 9.30% had positive C-reactive protein.

**Table 1:** Characteristics of patients with ulcerative colitis.

Characteristic		UC (n = 43)	
		Mean	SD
Age		34.5	11.0
BMI		25.0	4.89
Disease duration (yr)		4.87	4.14
IFX duration (wk)		114.12	85.34
Gender	Male n (%)	26 (60.5%)	
	Female n (%)	17 (39.5%)	
Azathioprine n (%)		41 (95.35%)	
Mesalamine n (%)		40 (93.02%)	
CRP positive n (%)		4 (9.30%)	
Disease Activity	Remission	17 (39.54%)	
	Active	26 (60.46%)	

CRP = C-reactive protein, SD = standard deviation, UC = ulcerative colitis.

The data presented in Table 2 demonstrate that the FCGR3A (rs396691) genotype was significantly linked to differences in infliximab levels and antibody response. Notably, the proportion of patients failing to achieve the therapeutic target trough level (≥3 μg/mL) varied significantly across genotypes (*P* = .005). All individuals with the AC genotype exhibited subtherapeutic infliximab levels, contrasting sharply with 55.6% of AA and 44.4% of CC genotype carriers who fell below the target. Pairwise comparisons further confirmed a significant disparity between the AA and AC groups (adjusted *P* = .032), underscoring the impact of the AC genotype on reduced drug exposure.

**Table 2:** IFX level and ATI (free and total) for patients with ulcerative colitis across rs396991 genotypes.

Variables			CC (n = 9)		AA (n = 19)		AC^[15]^		*P* value
IFX target (≥3 µg/mL)	Below target TL		4 (44.4%)		15 (78.9%)		15 (100%)		.005*^,^^a^
	Achieved target TL		5 (55.6%)		4 (21.1%)		0 (0.0%)		
Free ATI	Positive		1 (11.1%)		1 (5.3%)		3 (20.0%)		.41^a^
	Negative		8 (88.9%)		18 (94.7%)		12 (80.0%)		
Total ATI	Positive		2 (22.2%)		2 (10.5%)		8 (53.3%)		.02*^,^^a^
	Negative		7 (77.8%)		17 (89.5%)		7 (46.7%)		
IFX level (µg/mL)	Median (µg/mL)		3.41		1.09		0.34		.022*^,^^b^
	IQ		0.91–4.92		0.37–2.54		0.15–0.96		
Free ATI level (ng/mL)	Median (ng/mL)		47.93		36.35		49.47		.241^b^
	IQ		36.56–85.59		16.77–53.11		22.16–135.53		
Total ATI level (AU/mL)	Median (ng/mL)		4.04		4.8		15.55		.416^b^
	IQ		2.86–9.59		1.59–5.77		2.51–28.14		
Pairwise comparison				AA–CC		AC–CC		AA–AC	
IFX level (µg/mL)		Test statistic		−4.295		13.511		9.216	
		Unadjusted *P* value		.398		.011		.034	
		Adjusted *P* value c		1.0		.032*		.101	
IFX target		Fisher’s exact *P* value c		.097		.003*		.113	
Total ATI		Fisher’s exact *P* value c		.574		.210		.0098*	

ATI = anti-infliximab antibody, IFX = infliximab, IQ = interquartile range, TL = trough level.

a Chi-square.

b Kruskal–Wallis test.

c Bonferroni correction.

* Significant *P* < .05.

Consistent with these findings, median infliximab trough concentrations were significantly lower in the AC group (0.34 μg/mL) compared to the AA (1.09 μg/mL) and CC (3.41 μg/mL) groups (*P* = .022). This suggests that the AC genotype may be linked to increased drug clearance or diminished therapeutic response.

In terms of immunogenicity, the frequency of total ATI positivity was significantly elevated in AC genotype carriers (53.3%) relative to AA (10.5%) and CC (22.2%) groups (*P* = .02). Although median ATI levels were higher in the AC group, this difference did not reach statistical significance (*P* = .416), indicating that the increased antibody presence within this group may not correlate directly with antibody titer magnitude. Free ATI levels and positivity did not differ significantly among genotypes.

**Figure 1.**
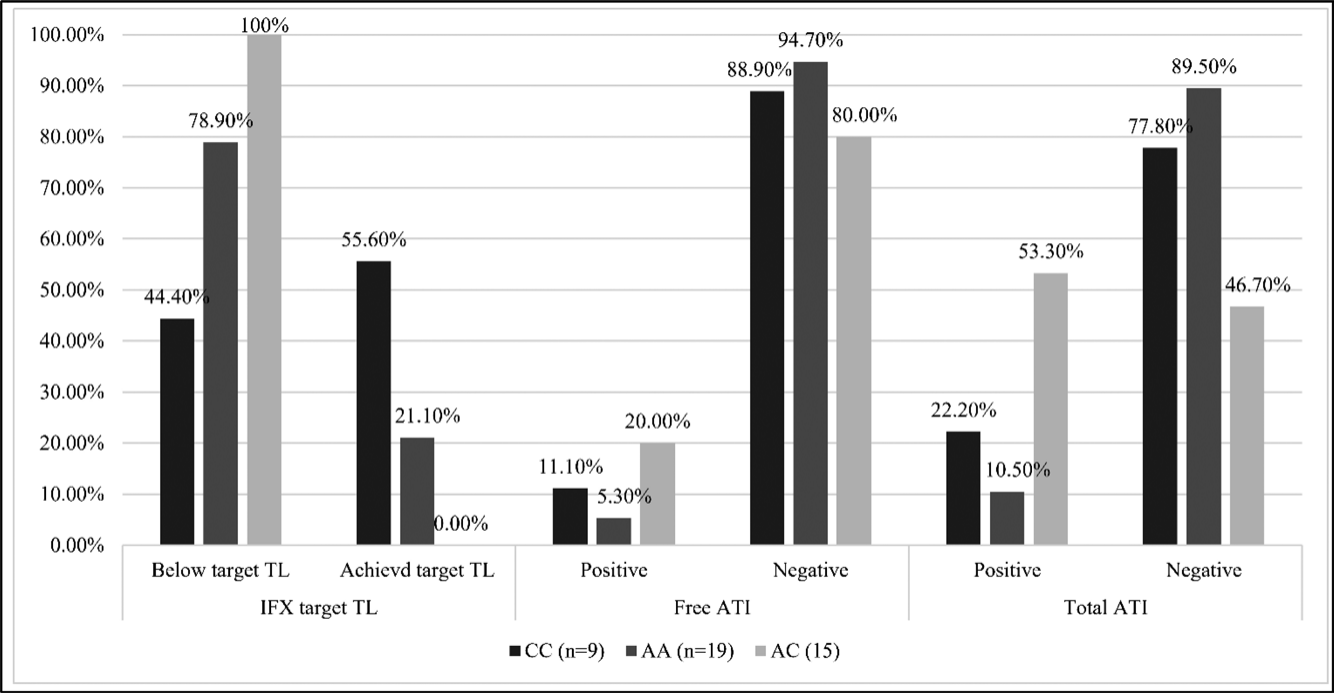
Distribution of infliximab trough levels (IFX TL) and anti-infliximab antibodies (ATI) across rs396991 genotypes (AA, AC, and CC) in ulcerative colitis patients.

As demonstrated in Figure 1, the below-target IFX TL was most frequent in AC (100%) and AA (78.9%) compared to CC (44.4%), while target TL was achieved most in CC (55.6% vs 21.1% AA, 0% AC). Free ATI positivity was rare, highest in AC (20%), with much lower rates in CC (11.1%) and AA (5.3%). For total ATI, positivity was most frequent in AC (53.3%), while the majority of CC (89.5%) and AA (77.8%) were negative. These findings suggest poorer IFX trough achievement and higher immunogenicity in AC carriers as compared to CC.

The multivariant logistic regression analysis as demonstrated in Table 3, that UC patients carrying the CC genotype have marginally significant increased odds (OR = 14.77, *P* = .050) of maintaining therapeutic IFX TL compared to the AA genotype, suggesting a favorable genetic influence on drug pharmacokinetics. Although antibody negativity showed a protective trend in this genotype, it was not statistically significant.

**Table 3:** Multivariant logistic regression analysis of genotype associations with infliximab trough levels and antibody status in ulcerative colitis.

Genotype comparison	Predictor	Estimate (B)	Lower 95% CI	Upper 95% CI	SE	Z	*P* value	Odds ratio
CC vs AA	Intercept	3.08	−2.33	8.48	2.76	1.12	.27	21.70
	IFX level (achieved vs below target)	2.69	0.00	5.39	1.38	1.96	.050*	14.77
	Free ATI (negative vs positive)	−1.39	−4.81	2.04	1.75	−0.79	.43	0.25
	Total ATI (negative vs positive)	−2.69	−7.12	1.73	2.26	−1.19	.23	0.07
	Age	0.03	−0.07	0.14	0.05	0.63	.53	1.03
	Gender (male vs female)	−1.03	−3.60	1.54	1.31	−0.79	.43	0.36
	Disease duration (mo)	−0.04	−0.08	0.00	0.02	−1.82	.07	0.96
	Azathioprine (yes vs no)	1.12	−2.72	4.96	1.96	0.57	.57	3.08
	Mesalamine (yes vs no)	−2.80	−8.33	2.73	2.82	−0.99	.32	0.06
AC vs AA	Intercept	5.33	−0.19	10.86	2.82	1.89	.06	207.00
	IFX level (achieved vs below target)	−10.39	−10.39	−10.39	0.00	−4.9E6	<.001*	0.00
	Free ATI (negative vs positive)	−0.66	−3.43	2.11	1.41	−0.47	.64	0.52
	Total ATI (negative vs positive)	−3.50	−6.65	−0.34	1.61	−2.17	.030*	0.03
	Age	−0.01	−0.11	0.09	0.05	−0.22	.82	0.99
	Gender (male vs female)	−1.25	−3.24	0.75	1.02	−1.22	.22	0.29
	Disease duration (mo)	−0.01	−0.03	0.01	0.01	−0.99	.32	0.99
	Azathioprine (yes vs no)	−0.02	−4.12	4.08	2.09	−0.01	.99	0.98
	Mesalamine (yes vs no)	−18.66	−18.66	−18.66	0.00	−98E5	<.001*	0.00

ATI = antiinfliximab antibody, IFX = Infliximab.

* Significant *P* < .005.

Conversely, the AC genotype is strongly linked with a higher likelihood of anti-infliximab antibody positivity, reflected by a markedly reduced odds of antibody negativity (OR = 0.03, *P* = .030). This implies that AC carriers are more prone to immunogenicity, which can diminish treatment efficacy. The model also produced extreme coefficient estimates for variables such as IFX achieving target level and mesalamine use within the AC group, indicating potential instability due to sparse data.

Other examined factors, including age, gender, disease duration, and concomitant use of Azathioprine or mesalamine, did not exert statistically significant effects on treatment outcomes across genotypes.

## 4. Discussion

This study provides an analysis of the associations between specific genotypes (rs396991) for UC, IFX TL, and antibody status (free and total anti-infliximab antibodies, ATI) in patients with UC. The results highlight significant variability in treatment outcomes based on genetic profiles, underscoring the potential role of pharmacogenomics in optimizing biologic therapy.

The primary clearance of these drugs occurs through the reticuloendothelial system, which involves 2 receptors with opposing roles. The FCGR receptor on endothelial reticuloendothelial system cells protects IgG from degradation, thereby extending its half-life. In contrast, Fc-gamma receptors on macrophages, natural killer cells (NK), and neutrophils promote the degradation of the IgG-FCGR complex in lysosomes. This process facilitates antigen presentation via major histocompatibility complex class II, increasing the likelihood of anti-IgG antibody production. Notably, a functional FCGR3A polymorphism (V158F), which alters antibody binding affinity, has been linked to infliximab response in CD.^[16]^

The FCGR3A-158V (valine) allotype has a higher affinity for IgG1 than the FCGR3A-158F (phenylalanine) allotype, and NK cells from V/V (CC).

Subjects are more potent in antibody-dependent cell-mediated cytotoxicity at low concentrations of antibody.^[20]^

It was found that NK cells from donors with the FCGR3A (rs396991) AA genotype exhibited significantly greater binding affinity to infliximab compared to those with the CC genotype. Additionally, peripheral blood mononuclear cells from AA genotype donors showed significantly higher infliximab-mediated antibody-dependent cell-mediated cytotoxicity than cells from CC donors^[11]^

Our result support favorable IFX kinetic and immunological outcomes in CC genotype which came along with previous study in Belgium, as researchers identified a significant pharmacogenetic association related to infliximab treatment in patients with CD. The findings suggested that FCGR and, potentially, antibody-dependent cell-mediated cytotoxicity play a role in the mechanisms of action of infliximab in CD. Specifically, the study highlighted that patients carrying the FCGR3A-158 CC genotype exhibited enhanced biological responses and, potentially, improved clinical outcomes to infliximab therapy. This marked the first time such a pharmacogenetic relationship was documented in the context of infliximab treatment for CD.^[16]^

A Japanese study reported a significant association between the FCGR3A AC polymorphism (rs396991) and clinical response to infliximab at 22 weeks in patients with rheumatoid arthritis, indicating that this genetic variant may serve as a predictive marker for primary infliximab response in this population. Specifically, the valine to phenylalanine (C to A allele) substitution in the gene encoding the FCGR3A receptor was associated with a higher likelihood of a favorable response to TNF-alpha inhibitors in the treatment of rheumatoid arthritis.^[21]^

In this study, the patients with CC and AA genotype found to have the lowest ATI compared to AC genotypes. In contrast to a recent study involving 103 patients with IBD found no significant association between ADA occurrence or rs396991 genotype and disease subtype or the specific anti-TNF agent used. However, the CC genotype was significantly correlated with increased ADA production (37.5% in CC vs 10.6% in AC and 5% in AA; *P* = .004), and was identified as an independent predictor of ADA formation in multivariate analysis. Additionally, patients with the CC genotype were more likely to require dose intensification of anti-TNF therapy (*P* = .03). These findings suggest that the rs396991 polymorphism influences ADA development and may help guide anti-TNF dosing and selection strategies in the management of IBD.^[15]^

However, another study found no association between the FCGR3A-158 polymorphism and clinical response to infliximab in CD, suggesting that this genetic variant may not directly affect patient management. However, consistent with earlier reports, the polymorphism appears to modulate the degree of C-reactive protein reduction in these patients.^[22]^

The clinical significance of these pharmacogenetic associations aligns with established literature, which demonstrates that detectable trough serum infliximab concentrations predict superior clinical outcomes in UC.^[23]^ Previous meta-analyses have shown that ATI presence is associated with a 3.2-fold increased risk of loss of clinical response to infliximab in inflammatory bowel disease patients.^[24]^ Our genetic findings provide mechanistic insight into this variability, suggesting that the rs396991 genotype may serve as a predictive biomarker for optimizing infliximab response.

The median infliximab levels observed across genotypes (CC: 3.14 μg/mL, AA: 1.09 μg/mL, AC: 0.34 μg/mL) demonstrate clinically meaningful differences, with the CC genotype achieving levels within the therapeutic range typically associated with favorable outcomes.^[25]^ This pharmacokinetic advantage, coupled with reduced immunogenicity, suggests that patients with the CC genotype may require standard dosing regimens. In contrast, those with AC or AA genotypes might benefit from intensified treatment strategies or alternative therapeutic approaches.

These findings complement recent pharmacogenetic research, which has identified variants in genes such as FCGR3A and TNFRSF1B that influence the anti-TNF response.^[13,26]^ The rs396991 variant adds to the growing body of evidence supporting personalized medicine approaches in inflammatory bowel disease management, potentially enabling clinicians to optimize treatment selection and dosing strategies based on genetic profiles.

Mechanistically, FCGR3A encodes the FcγRIIIa receptor, which is involved in the clearance of IgG immune complexes and antibody-dependent cellular cytotoxicity. Studies have shown that the V allele (valine at position 158) enhances binding affinity to IgG1 antibodies such as infliximab, potentially increasing clearance and promoting ADA development. This is supported by in vitro data demonstrating higher infliximab binding and antibody-dependent cytotoxicity in cells from individuals with the CC genotype.^[14]^ Clinically, this translates into increased infliximab elimination rates and a higher risk of relapse after drug discontinuation in patients with the CC genotype, especially when baseline inflammation is elevated.^[27]^

Meta-analyses and systematic reviews further confirm the link between FCGR3A polymorphisms and anti-TNF treatment outcomes in IBD, including UC. Although some studies have shown inconsistent results regarding clinical response, the consensus suggests that FCGR3A variants mainly influence biological response markers, such as decreases in C-reactive protein and ADA formation, rather than direct clinical remission rates.^[28]^ This indicates that FCGR3A genotyping could be especially helpful in predicting pharmacokinetic and immunogenicity profiles, thereby informing personalized dosing and treatment plans in UC. Since the LOR issue, in addition to the high cost of the medication.^[29]^

On the other hand, the protective effect of immunomodulator therapy against ATI formation has been well-established in the literature, with current combination therapy users showing significantly lower risk of developing ATI (hazard ratio 0.21; 95% CI 0.05–0.78; *P* = .02) compared to immunomodulator-naïve patients. Meta-analyses have demonstrated that combination therapy reduces ATI formation by approximately 50%, while simultaneously improving infliximab persistence and clinical outcomes. However, our findings suggest that this protective effect may vary between disease subtypes, with CD patients potentially deriving greater benefit from combination strategies.^[30,31]^ However, in this study despite majority of the patients revived immunomodulators there was still presence of ATI which effects on the IFX TL. Extreme values in regression, like those for mesalamine, often result from small or unbalanced data groups causing perfect prediction of outcomes. This leads to unstable, inflated coefficient estimates.

Polymorphism in FCGR3A has a significant impact on infliximab pharmacokinetics, immunogenicity, and treatment outcomes in patients with UC. By integrating FCGR3A genotyping into clinical practice, we could personalize therapy more effectively, identifying patients who are at higher risk for developing ADA and experiencing inadequate drug levels. This strategy has the potential to enhance long-term disease control and reduce the chances of treatment failure. However, further large-scale prospective studies in diverse UC populations, including those from Iraq, are necessary to validate these findings and refine genotype-guided management strategies.

### 4.1. Limitation

The generalizability of this study is limited by its small sample size and single-center design. The limited sample size might effect some of the result of multivariant regression and result, this leads to some unstable, inflated coefficient estimates. Moreover, the lack of follow-up data precludes evaluation of long-term outcomes and treatment durability. Further multicenter studies with larger cohorts, along with longitudinal follow-up, would be beneficial for confirming these findings.

## 5. Conclusion

In conclusion, the results show that the CC genotype of rs396991 is significantly associated with higher rates of achieving target IFX TL, lower total anti-infliximab antibody positivity, and higher median infliximab concentrations in patients with UC, compared to the AC and AA genotypes (*P* < .05 for all). Logistic regression confirms these associations, with the CC genotype showing increased odds of favorable pharmacokinetic and immunogenicity outcomes. In contrast, the AC genotype is associated with higher total ATI positivity and a failure to reach target infliximab levels.

## Author contributions

**Conceptualization:** Ahmad K. Al-Jalehawi, Samer Imad Mohammed.

**Data curation:** Ahmad K. Al-Jalehawi.

**Formal analysis:** Samer Imad Mohammed.

**Investigation:** Ahmad K. Al-Jalehawi.

**Methodology:** Samer Imad Mohammed.

**Software:** Ahmad K. Al-Jalehawi.

**Supervision:** Samer Imad Mohammed.

**Validation:** Samer Imad Mohammed.

**Writing – original draft:** Ahmad K. Al-Jalehawi.

**Writing – review & editing:** Samer Imad Mohammed.
